# Exploring rural Nurses' preparedness and post‐resuscitation experiences. An ethnographic study

**DOI:** 10.1111/jan.16295

**Published:** 2024-06-24

**Authors:** Katherine Riley, Rebekkah Middleton, Luke Molloy, Val Wilson

**Affiliations:** ^1^ School of Nursing University of Wollongong Wollongong New South Wales Australia; ^2^ Prince of Wales Hospital, South Eastern Sydney Local Health District & Ingham Institute Sydney New South Wales Australia

**Keywords:** compassion fatigue, ethnography, preparedness, resuscitation, rural nursing, training and education

## Abstract

**Aim:**

The focus of this paper is to provide a detailed ethnographic exploration of rural nurses' experiences of their resuscitation preparedness and the subsequent post‐resuscitation period.

**Design:**

An ethnographic study across two small rural hospital sites in New South Wales, Australia.

**Methods:**

Fieldwork was undertaken between December 2020 and March 2022 and included over 240 h of nonparticipant observation, journalling and interviews. Data were analysed using reflexive thematic analysis.

**Results:**

The first key theme—‘Sense of Preparedness’—included three subthemes: ‘Gaining experience’, ‘Issues with training and education’ and ‘Lack of warning’. The second key theme ‘Aftermath’ comprised two subthemes: ‘Getting on with it’ and ‘Making sense of the resus’.

**Conclusion:**

This study has highlighted the intricate relationship between resuscitative preparedness and the post‐resuscitation period in shaping rural nurse's experiences and their well‐being. Rural nurses are asking for an authentic and contextually relevant training experience that mirrors the unique rural challenges they experience. In the absence of frequent resuscitation presentations, the post‐resuscitation period should be viewed as a crucible moment that can be leveraged as a valuable learning opportunity enhancing rural nurses' sense of preparedness and the provision of quality resuscitation care.

**Impact:**

Having a greater level of insight into the challenges that rural nurses experience in the pre‐ and post‐resuscitation period is critical. This insight opens the door for fortifying policies and work processes that will better support rural nurses in the resuscitation environment.

**Reporting Method:**

Reporting complied with COREQ criteria for qualitative research.

**No Patient or Public Contribution:**

This study explored the experiences of rural nurses. No patient data were collected.

## 1 INTRODUCTION

Nurses working in small rural acute care settings are often described as rural ‘generalists’ due to their broad clinical skillset and their ability to care for a diverse range of patients including patients requiring resuscitation (Hendrickx & Winters, 2017). Resuscitations are considered a highly challenging aspect of clinical care requiring exceptional recall and application of technical knowledge (Yamada et al., 2018), meshed with non‐technical skills such as communication, leadership, teamwork and situational awareness (Gabr, 2019) during a time limiting high stakes environment. Despite rural emergency departments encountering fewer presentations of critically unwell patients (Sedgwick & Pijl‐Zieber, 2015), rural nurses have a responsibility to maintain and use advanced practice skills, ensuring that timely competent resuscitative care is delivered amid staffing and resource constraints (Riley et al., 2024). Rural nurses, as key stakeholders in the continuum of resuscitative care, must achieve proficiency in this area while navigating their preparedness for resuscitations and the subsequent transition back to their original roles in the post‐resuscitation phase.

The literature lacks adequate exploration of the rural resuscitation context, influenced by urban‐centric perspectives in the development of resuscitative practices (Armstrong et al., 2021; Clements et al., 2015; Jones et al., 2008). Rural nurses work in unique circumstances, characterized by limited medical presence, professional and geographical isolation, and an extended scope of practice (Burrows et al., 2019; Whiteing et al., 2022). This paper is part of a larger ethnographic study examining the resuscitation experiences and practices of rural nurses in Australia. This paper focuses on a crucial component of the overall study, centring on the contextual practices and norms that shape a rural nurse's preparedness for a resuscitation, and their approaches to navigating the post‐resuscitation period. This is essential for a holistic awareness of rural nurses' experiences of the resuscitation process.

## 2 THE STUDY

### 2.1 Aim

The focus of this paper is to provide a detailed ethnographic exploration of rural nurses' experiences of their resuscitation preparedness and the subsequent post‐resuscitation period.

### 2.2 Design

This study adopted an interpretivist paradigm and ethnographic methodology to address the overall study aims. Interpretivism provided a theoretical lens for understanding the subjective meaning of social phenomena and human behaviour within the rural resuscitation context from the perspective of rural nurses (Creswell, 2014). Interpretivism acknowledges that truth and knowledge are subjective, shaped by people's experiences, cultural backgrounds and historical contexts (Ryan, 2018). Therefore, interpretivist paradigm would argue that the behaviours, beliefs and practices adopted by rural nurses in the context of resuscitation are socially constructed (Pulla & Carter, 2018).

As a mode of inquiry, ethnography has been widely used in healthcare research, when observing and interacting with people in their own local context (Fetterman, 2020). Through this approach, we gain valuable insight into rural nurses' cultural practices, experiences, beliefs, norms and feelings associated with resuscitations in their rural department (Hammersley & Atkinson, 2007). An interpretivist paradigm complements the ethnographic approach, as both seek to understand the social world through immersion within specific cultural contexts, uncovering shared beliefs and practices of the cultural group being studied (O'Reilly, 2012). Thereby, enabling the researcher to explore areas such as tacit knowledge, practices and norms that prove challenging to capture through conventional questionnaires or surveys (Roper & Shapira, 2000).

### 2.3 Study setting and recruitment

The study was undertaken in two small rural emergency departments in New South Wales, Australia. Both emergency departments had level two emergency services. In NSW, emergency departments are assigned a level grade 1–6, whereby the lowest level indicates a small service that has limited workforce and service requirements compared to level 6 (a large trauma or referral emergency department) that requires the most complex services (NSW Ministry of Health, 2021).

#### 2.3.1 Sample

The sampling of nurse participants occurred in two ways. Firstly, ‘Key informants’ were purposively chosen due to their extended knowledge and experience of the rural resuscitation context (Polit & Beck, 2017). Key informants were often nurses who had recently undertaken a resuscitation and were willing to discuss their experiences. The nurse manager with insight into the activities of the department and their staff would also help identify key informants. Purposive sampling is ideally used in studies that require an in‐depth understanding of the phenomenon (Etikan et al., 2016), in this case the resuscitation experiences of rural nurses. This method allows the researcher to identify and deliberately select the participants who will provide information‐rich data (Welch, 2014), thereby assisting the researcher to understand cultural patterns inherent within their setting (Pahwa et al., 2023). Secondly, snowball sampling was a beneficial strategy when recruiting rural nurses, as leveraging referrals from key informants established a foundation of trust, significantly enhancing the interview process (Noy, 2008). Inclusion criteria for the study were any nurses that were clinically involved in resuscitations. All participating nurses had experienced a resuscitation event within the previous 6 months. Criteria that excluded participants included any other health professional and nursing staff not involved in resuscitations. There were 17 rural nurse participants interviewed. See Table 1 for participant demographics.

**TABLE 1:** Participant demographics.

Participant	Years of rural emergency experience	Extended scope of practice	Hospital site
Stella	<1 year	No	Site 1
Christine	1 year	No	Site 1
Ava	1 year	No	Site 2
Maria	2 years	No	Site 2
Bill	2.5 years	Yes	Site 1
Brian	3 years	Yes	Site 1
Luke	3 years	Yes	Site 2
Rachel	4 years	Yes	Site 1
Daniel	4 years	Yes	Site 1
Tracey	5 years	Yes	Site 1
Sophie	6 years	Yes	Site 1
Aaron	9 years	Yes	Site 2
Rana	>10 years	Yes	Site 2
Bridget	>10 years	Yes	Site 1
Jane	>10 years	Yes	Site 2
Kim	>10 years	Yes	Site 1
Ally	>10 years	Yes	Site 1

### 2.4 Data collection

The primary investigator completed the fieldwork at both sites. Site 1 fieldwork occurred during COVID‐19 pandemic, from December 2020–April 2021, while site 2 fieldwork occurred during October 2021–March 2022. During these periods, COVID‐19 restrictions were in place in the hospitals. Data collection was minimally affected during site 1 fieldwork, and mask wearing was a requirement of entry into the department. Site 2 fieldwork was halted mid‐way through for 1 month, and this was due to an increase in COVID‐19 transmissions in NSW. In line with an ethnographic approach, multiple methods were undertaken during fieldwork (Fetterman, 2020), including, observation of nursing practice, interviews (semi‐structured/unstructured) and participant journaling. A total of 240 h of non‐participant observation was completed by the first author equally across the two rural sites covering a variety of shifts and days. This approach provided a holistic insight into the ebbs and flow of a rural emergency department and included observing resuscitations, nurse–patient and nurse–nurse/health professional interactions, clinical handovers/huddles and interactions with staff. Observation in the field enabled the researcher to experience the everyday routines and practices of rural nurses and how these impacted on resuscitation.

During observation periods in the clinical environment, informal conversations were undertaken between the researcher and participants, offering a valuable opportunity to build rapport and validate real‐time observations (Swain & King, 2022). Data obtained from these interactions were documented contemporaneously in written or audio fieldnotes and included reflections about conversations and practices. Interviews were also performed away from the clinical area, such as an empty office space. This was to ensure that the participants had a quiet space to reflect and to ensure privacy and confidentiality of the discussion were maintained (Knott et al., 2022). The interviews were semi‐structured in nature and audio recorded (see Table 2). Interviews were 20–60 min in length (mean = 38 min.). The first three interviews were transcribed verbatim by the author, the remainder were transcribed by a transcription service and checked against the original recordings (step 1) (Braun & Clarke, 2022). Only one nurse opted to write in a participant journal of their resuscitation experience, which was then uploaded into NVIVO in preparation for coding.

**TABLE 2:** Example of semi‐structured interview questions.

Please describe your last resuscitation
What helps you feel more prepared for a resuscitation?
Please tell me about any resuscitation training and education you have received
What happens after a resuscitation?
What does a good resuscitation look like?

### 2.5 Data analysis

Data analysis began during fieldwork and adopted a reflexive thematic approach. A systematic six‐step data analysis method, as outlined by Braun and Clarke (2022), guided the researchers and involved comparing emerging findings between observation, participant journal and interview data. To support the management of data, NVIVO 12 software was used. The first author undertook the original coding of the data using NVIVO (step 2), while third author blindly coded for cross‐checking. Once codes were developed, first author began generating themes with the use of mind mapping (step 3). The research team worked together to review, refine and revise the final themes based on the codes (step 4–5), in preparation for writing up a richly contextual and interpretative narrative (step 6) (Braun & Clarke, 2022). A reflexive journal was kept by the first author, encouraging reflexivity and to mitigate the influence of the researcher's positionality including prior knowledge, past resuscitation experiences and internalized beliefs and assumptions (Adler, 2022; Higginbottom et al., 2013).

### 2.6 Ethical considerations

Ethics approval was obtained by Joint University of Wollongong and Illawarra Shoalhaven Local Health District Health and Medical Human Research Ethics Committee (2019/PID15431) on the 15 October 2020. Informed consent was required from both nursing staff and patients. As a result, many resuscitations were not observed due to the ethical issue associated with a patient's capacity to consent and time constraints. Patients that require resuscitation lack the decision‐making capacity to provide informed consent due to factors such as their level of consciousness, hypoxia and medications. In addition, many patients did not have sufficient time to carefully weigh up the risks and benefits of their involvement; therefore, they were excluded from observation.

### 2.7 Trustworthiness

To ensure the study met the trustworthiness criteria (credibility, transferability, dependability and confirmability) as outlined by Lincoln and Guba (1985), a range of practices were adopted. Methodological and investigator triangulation was employed as a cross‐checking process to strengthen the study's credibility and confirmability (Stahl & King, 2020). Multiple data collection methods (observation, interviews and participant journaling) were used which allowed for validation of findings. The research team members reviewed fieldnotes, interviews and contributed to the coding and theming of the data. The use of ‘thick description’ of participants’ narratives about their experiences of resuscitations enhanced transferability (Lincoln & Guba, 1985). The primary investigator conducted member checking as a measure to enhance the study's credibility (Shenton, 2004). Six participants contributed to the validation process, by reviewing themes, allowing them to confirm whether they recognized their experiences in the themes (Harvey, 2015). The primary investigator, who has been a rural emergency nurse, maintained a reflexive approach throughout the study. This involved evaluating their insider/outsider role, relationships and assumptions using a reflexive journal and engaging in regular discussions with the research team to ensure transparency and awareness (Braun & Clarke, 2022).

## 3 FINDINGS

Two key themes and five sub‐themes were identified, exploring the contributing factors that influence a rural nurse's experience and practice of resuscitation (See Table 3). All participants were de‐identified and assigned pseudonyms. Quotes are used to illustrate key points and are drawn from interviews (I), fieldnotes of observation (F) and staff reflective journal (RJ).

**TABLE 3:** Key themes and sub‐themes.

Key themes	Sub‐themes
Sense of preparedness	Gaining experience Issues with training and education Lack of warning
Aftermath	Getting on with it Making sense of the resus

### 3.1 Sense of preparedness

The ‘sense of preparedness’ among rural nurses referred to the intrinsic confidence they held regarding their readiness to manage a resuscitation. Rural nurses described three subthemes that functioned as barriers to their overall ‘sense of preparedness’; these included, gaining experience, issues with training and education and lack of warning.

#### 3.1.1 Gaining experience

Rural nurses regarded resuscitative experience essential for fostering a sense of preparedness. However, they encountered hurdles in acquiring experience—*‘It takes a lot longer, you don't see the same acuity in a little place as you would in a big place, and not as often’* (Aaron). Many experienced rural nurses believed that ‘junior’ nurses should seek experience elsewhere first so they can have a greater exposure to the problem‐solving and teamwork dynamics inherent in resuscitation environments.

I think some of the junior nurses really need to go off and have that base experience, I mean simulation might help, but it's just the real‐life stuff where experience is going to help. Working rural, you do not have resus's all the time. I was in the larger hospital for 11 years, there was a resus nurse, you were allocated that job, you'd get a resus one, two, three times a week on your shifts. It was just a bit more routine and regular. Whereas here, it's not like that at all. (I)‐Kim

While gaining experience at a larger hospital may offer less experienced staff increased exposure to resuscitations, the practical outcome did not consistently translate into meaningful experiences that supported them in a rural setting.

I did all my training up [north], in a resus up there they have a whole big team come and rush to your bedside. There's ICU doctors and everyone, there's so many people there….then come to a rural hospital and I have, not the least experience [in rural resuscitation]. (I)‐Stella

In larger hospitals, designated medical emergency teams often support wards/units by providing an experienced team to lead and manage the resuscitation. While in rural sites, ward staff with basic life support skills were called upon to assist because they were readily available on short notice. However, they usually lacked resuscitative experience. ‘So, the ward is aged care. We don't have any resus's, because everyone's NFR (Not For Resuscitation). So, we don't get much experience.’ (I)‐Maria. Inexperienced nurses frequently felt unprepared and voiced apprehensions regarding their limited experience in resuscitation when asked to help, especially those who worked in low acuity areas such as the general wards or adjacent aged care facility. Stella, a ward nurse, provides insight into this experience after being called to the emergency department to assist in a resuscitation.

It was an afternoon shift, we [the ward] already had five patients each. I probably should have spoken up and said ‘no, I want someone else to show me through the scribe role’ because it was my first resus. I didn't think that that was an option, we were short on the ward, and they needed a person in resus. (I)‐Stella

This situation had a ripple effect in the resuscitation room. Emergency staff were often unaware of the ward nurses' level of resuscitation experience; this sometimes led to some ward staff feeling pressured to work beyond their scope of practice or discrepancies in their role performance.

She [Stella] just wrote everything she saw. Normally they do education for the scribe role, but she hadn't done the education. I had no idea, not until after when she burst into tears. It is a coroner's case, so I had to spend hours reviewing the notes. (I)‐Ally

While having resuscitative experience is an important skill in rural settings, the ongoing challenge for rural nurse managers was recruiting experienced staff to the department. Skilled rural agency nursing staff temporarily filled vacancies, meeting the short‐term needs of the department. However, the long‐term stability of the department is dependent on recruiting and retaining an experienced team.

When placing an expression of interest out, Tanya often had new graduate nurses applying. Some may have completed an emergency department placement as a student, but Tanya prefers, they have experience in a rural ED. (F)

Recruiting nurses with rural emergency experience is preferred, as their adaptability and familiarity with the rural context and its processes, typically translated to lower reliance on additional support.

#### 3.1.2 Issues with training and education

Participants deemed resuscitative training and education essential for practice and significantly contributed to a sense of preparedness. When a rural nurse completed the required training and education, they had an increased scope of practice that enabled them to use skills necessary for resuscitation. For example, many of the participants working in the emergency department had undertaken resuscitation‐specific training such as Advanced Life Support (ALS) (Australian Commission on Safety and Quality in Health Care, 2021). However, many did not hold a relevant post‐graduate qualification or had successfully completed the First Line Emergency Care Course (FLECC). Successful completion of this course enabled rural nurses working in ED to work to the full scope of practice as outlined in the Adult and Paediatric NSW Rural Emergency Guidelines during a resuscitation (Agency for Clinical Innovation, 2022). With this scope of practice, rural nurses were able to assess and treat life‐threatening conditions such as cardio‐respiratory arrest, severe sepsis and trauma injuries such as burns/near drowning/head injuries (Agency for Clinical Innovation, 2022). Although this advanced training provided credentialing needed to extend their scope of practice, training was not always contextually fit for purpose.

Jane was concerned about sending her staff for ALS and FLECC training knowing that the simulation scenarios were based off larger hospitals staffing and resources. She stated, ‘The context doesn't match, it doesn't provide a rural perspective when sometimes you are the only person that's there, you're the first responder but you're everything. The simulation training doesn't match the rural resuscitative environment’. (F)

Additionally, nurses had to travel to a larger hospital, usually situated 1–2 h away from their rural communities, to take part in FLECC and ALS training. Chronic staff shortages plagued the role of nurse managers as they struggled to ensure that available nursing staff could replace staff attending the training days. This struggle often resulted in nurse managers working the shift themselves.

[Nurse managers] struggle to allocate/maintain training for staff or sending staff off to training like ALS because they've only got 10 staff on the roster, it's hard to maintain that sort of training when they've got to staff an ED and acute care ward 24/7. (F)

Rural nurses considered in‐hospital resuscitation training and education as beneficial if conducted regularly and included opportunities for interdisciplinary involvement. However, despite some training being ‘in‐house’ there were still hurdles in access, due to educator availability and resuscitation training that was often professionally siloed.

Talking to Sophie about resuscitation training, she mentions that once or twice a year, one of the doctors holds an education day where other doctors come, and the Dr was finding that no nursing staff were going. The Dr asked Sophie why nurses weren't interested, and she said because doctors are paid, and nurses aren't paid for their time. (F)

Preparedness extended beyond an individual's own sense of responsibility. Rural nurses expressed the importance of having all staff members trained in ALS, as this helped rural nurses feel more prepared in the event of a resuscitation. Highlighting that preparedness was a broader, collective issue.

A good resus is when you've got confidence in the people of the team, so Andrea and I would be good. Sara and Loretta are on this afternoon, I know they both know their ALS and they are good at keeping themselves up to date. (I)‐Jane

#### 3.1.3 Lack of warning

Preparing staff and resources for a resuscitation takes time. While having experienced nursing staff who are appropriately trained aids a nurse's sense of preparedness, this can be quickly eroded in situations where there is insufficient warning of a resuscitation – *‘it takes time to find people to help and all actions need to be done quickly’*. (RF) – Bridget. To circumvent this situation, the ambulance service phones through a ‘bat call’ to the emergency department notifying of an imminent emergency, such as a cardiac arrest. A ‘bat call’ is considered by rural nurses as the most effective way to be notified if a critically unwell person was being transported to the emergency department. Kim notes that '*when you are notified via a ‘bat call’ you have time to call the doctor in, get your team together'* (I); however, these calls were not always provided by the ambulance service. Notification via a ‘bat call’ not only allowed time for rural nurses to organize an action plan, to consider the ongoing staffing/patient needs for the emergency department, such as acuity of existing patients in department, skill mix, staff well‐being and the availability of a medical officer.

I think it was me, another nurse, and the doctor, just the three of us… there was only one other person left in the ED that stayed with the four‐bedder. We still had quite a few sick ones in the rest of our ED. It wasn't that you could really free up people. (I)‐Christine

In addition, both rural communities in this study had access to a local ambulance service during an emergency; however, many patients chose not to call an ambulance but rather self‐presented to the hospital.

I don't know why, but they (patient) are quite hesitant at calling the ambo, maybe because the ambo might prefer to transfer them to a more suitable destination for care…. because majority of the time we don't have a medical officer. (I)‐Luke

Similarly, when a patient was brought in via private car, rural nurses had no time to prepare staff, their personal protective equipment and resuscitation equipment; they also had no preliminary support from the ambulance service, who would have assessed and commenced treatment on the patient.

Somebody rings the doorbell, it was 11 o'clock at night…. there's no pre‐warning she's just at the front doorbell with a towel and she's got blood clots in it and she's retching. (I)‐Rana

In the absence of advance warning, rural nurses felt unprepared for a resuscitation, this had a flow on affect into their resuscitation practices and overall experience of the resuscitation event.

### 3.2 The aftermath

The post‐resuscitative period was a challenging time for rural nurses. It was expressed as a disconnect between the fast‐paced resuscitation and returning to the normality of caring for the less critically ill patients in the department.

#### 3.2.1 Getting on with it

After a resuscitation, rural nurses would resume the care of their patients. Some rural nurses found the transition back to care challenging for several reasons, such as feeling obligated to leave family shortly after the resuscitation and return to other patients, this often presented as a moral dilemma for nurses.

Most of the time I don't get to experience being able to be part of the family's experience, because I have to move on to see other patients. Ideally, you finish the resus, you've done everything you could, you can talk to the family and reflect and then go back to your work. Whereas most of the time you do a resus, and you go back to work. (I)‐Bill

Even after traumatic resuscitations such as ones involving paediatrics, rural nurses felt duty‐bound to return to their patients. Rana shares her experiences after resuscitating a child.

You know, you can have a terrible death, a terrible experience in ED; and you know, you're meant to just clean up the bed and go and empty a bed pan sort of thing. But it's just back to normal. I think that's awful, and it's hard to do, but it's what's expected. You don't go and have a cry in the corner or, you know, it's just, it's not what is expected. So, you just rally on. (I)‐Rana

Although returning to patient care after a resuscitation re‐establishes the staffing levels of the department, it can be unsettling for nurses who are still processing their emotions while simultaneously providing care to patients.

I've had experiences where, I guess I don't feel right for the rest of the shift. If someone comes in with a sore toe and they're saying it's the worst thing in the world, I wouldn't say I'm disappointed, but I just don't want to deal with that. It's like, you have no idea. But that lack of empathy for that patient is because I haven't dealt with my own emotional issues with what I just experienced in the resus. (I)‐Brian

#### 3.2.2 Making sense of the resus

‘Making sense of the resus’ was a strategy that many rural nurses described and involved a reflective process aimed to critique their individual and team performance during the resuscitation, as Ally explains ‘could we have done it better?’. (I). It also aided rural nurses in finding closure after a resuscitative event enabling them to cognitively and emotionally ‘move on’. The process was commonly undertaken over a broad timeline that occurred hours or days after the event. Making sense of the resus' was usually undertaken in solitude or involved informal chats with colleagues or family, spurred on by emotions such as anger, worry and doubt associated with the resuscitation.

The ones that make me the angriest or the ones I find very difficult to deal with are the ones where it didn't go as I think it should have gone. So, after a resus, I go off and I Google things, I go online I go looking through CINAHL and looking up articles about haemoptysis and whatever, learning because that's my way of going ‘well next time I will do this instead’ or ‘I did that alright that's good’. (I)‐Jane

Some rural nurses found comfort in talking with their own families about their experiences of the resuscitation, but this often did not resolve ruminating thoughts.

I had a chat to some family that night when I went home, but it took a few hours to get to sleep because my mind was just wandering and just thinking of it. If I did enough… Yeah, took a bit to get to sleep that night. (I)‐Stella

After a resuscitation, patients were transferred via retrieval helicopter or ambulance services to a larger referral hospital with a higher level of care. This signalled a limbo period for nurses, where the patient's condition is unknown, hampering their ability to process and make sense of the resuscitation. Daniel describes in an interview, ‘I was worried about him… yeah… wondering how he would have gotten on’. It also gave rise to concerns about whether they will be judged by the receiving hospital for the resuscitative care provided. Following up on the patients via online medical notes was another way nurses were able to ‘make sense’ of resuscitation as it alleviated their fear of being judged by the receiving hospital. Bridget describes,

I always follow up patients to ascertain if our clinical judgement/management plan were correct. There is always concern that our judgements will be questioned. (RJ)

When a patient's death was unexpected, nurses would experience conflicting thoughts and feelings about the resuscitation. ‘Making sense’ was often delayed until the patient's cause of death was known, or when they were able to talk to a trusted medical officer.

Unexpected, lack of control I'm not sure of the right word, like when the resus turns the other way, and then questioning if I did everything right. I felt better that I have spoken to Dr S (1 week post resus), I still feel I know you shouldn't say it but back of me thinks until I know the results – did she need to die? (I)‐Ally

Rural nurses question the adequacy of their care when the patient's outcome is unknown, accentuating the persistent burden rural nurses face in reconciling their actions in a rural setting where post‐resuscitation debriefing is limited.

## 4 DISCUSSION

This study highlights the intricate relationship that resuscitative preparedness and the post‐resuscitation period has on rural nurse's experience of resuscitation. Although there are many studies that have examined rural nurse preparedness in a range of contexts, such as emergency disaster management (Baack & Alfred, 2013; Brewer et al., 2020), there is little known about what contributes to a rural nurse's sense of preparedness for a resuscitation. This study is also the first that has sought to understand rural nurses' experiences during the post‐resuscitation period. Exploring both these themes in isolation of one another would negate the value that both bring in conceptualizing the causal influences shaping a rural nurse's experience of resuscitation. Together these themes provide a more holistic and nuanced understanding of the rural context in which resuscitations occur and how this may inform nursing practice.

One of the challenges that hindered a rural nurse's sense of preparedness was the inter‐relationship between resuscitative skills and limited exposure to or experience of resuscitations. This phenomenon is not new, with over two decades of literature detailing the ongoing struggles with maintaining resuscitative competence (Endacott & Westley, 2006; Finn, 1996; Sedgwick & Pijl‐Zieber, 2015; Zanno et al., 2022). While workforce and policy enhancements have been implemented to address issues such as ‘extended scope of practice’ guidelines (Cant et al., 2011) and the role of rural nurse practitioner (Jennings et al., 2021), rural nurses continue to encounter challenges in developing and sustaining competence in resuscitation. Externally sourced and onsite training and education often fell short in addressing the unique challenges of rural departments and the needs of rural nurses. Chronic staff shortages, limited educator hours and out‐of‐pocket expenses acted as significant barriers to accessing training. While these findings have been noted in previous studies, calling for an ‘out of the box’ stance to circumvent such barriers (Whiteing et al., 2022; Wolf & Delao, 2013), it is still evident that this has yet to reach the rural clinical environment.

What is evident is that rural nurses are asking for a more authentic and fit‐for‐purpose training experience. Rural healthcare settings are dynamic and unpredictable; therefore, resuscitation training and education needs to be responsive to those nuances. Given the ambiguity about the most effective training methods for rural nurses (Corner et al., 2023), adopting a co‐design approach during the development and implementation of rural resuscitation training could offer a novel solution to improve the applicability of training (Slattery et al., 2020). Such an approach would support the unique needs of each rural setting by ensuring that local knowledge of the rural context is championed, empowering rural nurses to actively participate in the conception and implementation. While this approach is absent in rural literature, it has been implemented successfully in other healthcare contexts such as the development of rapid response teams (Won & Kang, 2022) and nursing postgraduate programs (Theobald et al., 2020).

Rural nurses raised concerns about critically unwell patients self‐presenting to the emergency department and the subsequent chaos that ensued to meet the initial resuscitative needs of the patients. The underutilization of ambulance services poses significant risk to the health outcomes of rural communities (Reed & Bendall, 2015) and is reflected in the international literature, detailing that patients in rural areas are less likely to contact emergency services when critically unwell than their urban counterparts (Beillon et al., 2009; Sariyer et al., 2017). While the findings from this study provide insight into barriers that effect a rural nurse's sense of preparedness, further research is required to better understand underutilization of emergency services from the service user's perspective.

The aftermath of a resuscitation emphasized the lasting impact the event had on a rural nurses' capacity to transition back to their clinical roles, resulting in some nurses experiencing insomnia, ruminating thoughts and in some instances, a lack of empathy towards less critical patients. While these factors are consistent across a broad spectrum of emergency departments (Hao et al., 2023; Riley et al., 2022), the rural context offers a unique set of challenges that have been shown to affect the mental well‐being of rural nurses (Dekeseredy et al., 2019). Extensive research has established close links between compassion fatigue and the psychological burden stemming from resuscitation (Berg et al., 2016; El‐Ashry et al., 2023; Koželj et al., 2021). While resuscitations are known to amplify such symptoms, Caulfield et al. (2023) highlight that compassion fatigue is more likely compounded by a multitude of other factors, such as staff shortages, workload and poor rostering. Along with various stressors, this complexity may stem from the altruistic nature of the nursing profession, leading rural nurses to prioritize returning to patient care over addressing their own emotional needs following a resuscitation.

After a resuscitation, rural nurses often sought informal methods to make sense of their experiences, resorting to sources like Google, family and colleagues for validation. While these sources might offer anecdotal or experiential insights, they may not consistently align with best practice principles (Blomquist & Lasiter, 2022). Relying on non‐traditional sources might compromise the rigorous verification processes inherent in evidence‐based practices. Nilsen et al. (2017) report that barriers to integrating EBP frequently emerge from adaptive practices or ingrained habits formed from past experiences. Such barriers include time and resource constraints, or appropriate models for debriefing that meet the needs of the rural context (Floridis, 2023). In the absence of frequent resuscitation presentations, the post‐resuscitation period should be viewed as crucible moments that can be leveraged as learning opportunities when enhancing rural nurses' resuscitative knowledge and confidence (Bettinger et al., 2021; Floridis, 2023). This highlights the need for stronger organizational support to assist rural nurses in navigating the post‐resuscitative period, amid increasing workforce pressures.

### 4.1 Limitations

This study was the first to explore the experiences of rural nurses at two rural sites in New South Wales Australia. While two sites increase the rigour of the findings, the transferability of these findings may be limited to participants and settings similar to those studied. The first author's background as a rural emergency nurse provided insight into the field; however, this may have resulted in some rural nurses altering their interview responses or clinical practices. The first author purposively delayed interviews until rapport had been built with each participant, while data triangulation was used to validate the data.

## 5 CONCLUSION

This Australian study has provided insight into the difficulties rural nurses experience in resuscitation preparedness and the post‐resuscitation period and the impact this has on the provision of resuscitation care and a nurse's well‐being. It highlights the importance of context and accessibility when delivering fit‐for‐purpose resuscitation training. During the post‐resuscitation period, rural nurses grapple with heightened emotions with limited avenues to process, resulting in an increased psychological burden on nurses to prioritize caregiving over their own mental well‐being. With limited resuscitation exposure, rural nurses with organizational support can harness these experiences as invaluable learning opportunities to enhance their professional growth and preparedness for future resuscitations.

## AUTHOR CONTRIBUTIONS

Made substantial contributions to conception and design, or acquisition of data, or analysis and interpretation of data: KR, RM, VW LM. Involved in drafting the manuscript or revising it critically for important intellectual content: KR, RM, VW LM. Given final approval of the version to be published. Each author should have participated sufficiently in the work to take public responsibility for appropriate portions of the content: KR, RM, VW LM. Agreed to be accountable for all aspects of the work in ensuring that questions related to the accuracy or integrity of any part of the work are appropriately investigated and resolved: KR, RM, VW LM.

## CONFLICT OF INTEREST STATEMENT

No conflict of interest has been declared by the authors

## FUNDING INFORMATION

No funding was received for this study.

### PEER REVIEW

The peer review history for this article is available at https://www.webofscience.com/api/gateway/wos/peer‐review/10.1111/jan.16295.

## Data Availability

Research data are not shared.

## References

[jan16295-bib-0001] Adler, R. H. (2022). Trustworthiness in qualitative research. Journal of Human Lactation, 38(4), 598–602. 35929019 10.1177/08903344221116620

[jan16295-bib-0002] Agency for Clinical Innovation . (2022). *NSW rural adult emergency clinical guidelines* GL2022_004. https://www1.health.nsw.gov.au/pds/ActivePDSDocuments/GL2022_004.pdf

[jan16295-bib-0003] Armstrong, P. , Peckler, B. , Pilkinton‐Ching, J. , McQuade, D. , & Rogan, A. (2021). Effect of simulation training on nurse leadership in a shared leadership model for cardiopulmonary resuscitation in the emergency department. Emergency Medicine Australasia, 33(2), 255–261. 32856402 10.1111/1742-6723.13605

[jan16295-bib-0004] Australian Commission on Safety and Quality in Health Care . (2021). National safety and quality health service standards (2nd ed.). Australian Commission on Safety and Quality in Health Care.

[jan16295-bib-0005] Baack, S. , & Alfred, D. (2013). Nurses' preparedness and perceived competence in managing disasters. Journal of Nursing Scholarship, 45(3), 281–287. 23574544 10.1111/jnu.12029

[jan16295-bib-0006] Beillon, L. M. , Suserud, B. O. , Karlberg, I. , & Herlitz, J. (2009). Does ambulance use differ between geographic areas? A survey of ambulance use in sparsely and densely populated areas. The American Journal of Emergency Medicine, 27(2), 202–211. 19371529 10.1016/j.ajem.2008.01.012

[jan16295-bib-0007] Berg, G. M. , Harshbarger, J. L. , Ahlers‐Schmidt, C. R. , & Lippoldt, D. (2016). Exposing compassion fatigue and burnout syndrome in a trauma team: A qualitative study. Journal of Trauma Nursing, 23(1), 3–10. 26745533 10.1097/JTN.0000000000000172

[jan16295-bib-0008] Bettinger, K. , Mafuta, E. , Mackay, A. , Bose, C. , Myklebust, H. , Haug, I. , Ishoso, D. , & Patterson, J. (2021). Improving newborn resuscitation by making every birth a learning event. Children (Basel, Switzerland), 8(12), 1194. 34943390 10.3390/children8121194 PMC8700033

[jan16295-bib-0009] Blomquist, M. , & Lasiter, S. (2022). Nurses' coping strategies during and after an adult in‐hospital resuscitation attempt: A scoping study. Journal of Clinical Nursing, 31(17–18), 2437–2449. 35927889 10.1111/jocn.16128

[jan16295-bib-0010] Braun, V. , & Clarke, V. (2022). Thematic analysis: A practical guide. Sage.

[jan16295-bib-0011] Brewer, C. A. , Hutton, A. , Hammad, K. S. , & Geale, S. K. (2020). A feasibility study on disaster preparedness in regional and rural emergency departments in new South Wales: Nurses self‐assessment of knowledge, skills and preparation for disaster management. Australasian Emergency Care, 23(1), 29–36. 31926956 10.1016/j.auec.2019.12.005

[jan16295-bib-0012] Burrows, G. L. , Calleja, P. , & Cooke, M. (2019). What are the support needs of nurses providing emergency care in rural settings as reported in the literature? A scoping review. Rural and Remote Health, 19(2), 1–11. 10.22605/RRH4805 31088108

[jan16295-bib-0013] Cant, R. , Birks, M. , Porter, J. , Jacob, E. , & Cooper, S. (2011). Developing advanced rural nursing practice: A whole new scope of responsibility. Collegian, 18(4), 177–182. 22256558 10.1016/j.colegn.2011.08.001

[jan16295-bib-0014] Caulfield, R. , Wiseman, T. , Gullick, J. , & Ogilvie, R. (2023). Factors preceding occupational distress in emergency nurses: An integrative review. Journal of Clinical Nursing, 32(13–14), 3341–3360. 35871282 10.1111/jocn.16461

[jan16295-bib-0015] Clements, A. , Curtis, K. , Horvat, L. , & Shaban, R. Z. (2015). The effect of a nurse team leader on communication and leadership in major trauma resuscitations. International Emergency Nursing, 23(1), 3–7. 24880695 10.1016/j.ienj.2014.04.004

[jan16295-bib-0016] Corner, S. , Dahlke, S. , & Hunter, K. (2023). Educational needs of rural nurses when entering practice. Online Journal of Rural Nursing and Health Care, 23(1), 32–69.

[jan16295-bib-0017] Creswell, J. W. (2014). Research design: Qualitative, quantitative, and mixed methods approaches (4th ed.). SAGE Publications.

[jan16295-bib-0018] Dekeseredy, P. , Landy, C. M. K. , & Sedney, C. L. (2019). An exploration of work‐related stressors experienced by rural emergency nurses. Online Journal of Rural Nursing and Health Care, 19, 2–24.

[jan16295-bib-0019] El‐Ashry, A. M. , Elsayed, S. M. , Ghoneam, M. A. , & Atta, M. H. R. (2023). Compassion fatigue and stress related to cardiopulmonary resuscitation: A study of critical care nurses' experiences. BMC Nursing, 22(1), 482. 38110907 10.1186/s12912-023-01640-y PMC10726549

[jan16295-bib-0020] Endacott, R. , & Westley, M. (2006). Managing patients at risk of deterioration in rural hospitals: A qualitative study. Australian Journal of Rural Health, 14(6), 275–279. 17121508 10.1111/j.1440-1584.2006.00829.x

[jan16295-bib-0021] Etikan, I. , Musa, S. A. , & Alkassim, R. S. (2016). Comparison of convenience sampling and purposive sampling. American Journal of Theoretical and Applied Statistics, 5(1), 1–4.

[jan16295-bib-0022] Fetterman, D. M. (2020). Ethnography step‐by‐step (4th ed.). Sage Publishing.

[jan16295-bib-0023] Finn, J. (1996). The role of nurses in cardiopulmonary resuscitation and defibrillation. Collegian, 3(3), 31–34. 9265496 10.1016/s1322-7696(08)60178-x

[jan16295-bib-0024] Floridis, J. (2023). Debriefing after critical incidents in rural and remote healthcare settings‐a remote clinical perspective. Rural and Remote Health, 23(1), 1–4. 10.22605/RRH7450 36945148

[jan16295-bib-0025] Gabr, A. K. (2019). The importance of nontechnical skills in leading cardiopulmonary resuscitation teams. The Journal of the Royal College of Physicians of Edinburgh, 49(2), 112–116. 31188338 10.4997/JRCPE.2019.205

[jan16295-bib-0026] Hammersley, M. , & Atkinson, P. (2007). Ethnography: Principles in practice (3rd ed.). Routledge.

[jan16295-bib-0027] Hao, Y. , Zhu, W. , Wu, H. , Guo, Y. , Mu, W. , Li, D. , Ren, X. , & Fan, L. (2023). Experience of cardiopulmonary resuscitation by healthcare professionals in emergency departments: A descriptive phenomenological study. International Emergency Nursing, 70, 101336. 37657134 10.1016/j.ienj.2023.101336

[jan16295-bib-0028] Harvey, L. (2015). Beyond member‐checking: A dialogic approach to the research interview. International Journal of Research & Method in Education, 38(1), 23–38.

[jan16295-bib-0029] Hendrickx, L. , & Winters, C. (2017). Access to continuing education for critical care nurses in rural or remote settings. Critical Care Nurse, 37(2), 66–71. 10.4037/ccn2017999 28365651

[jan16295-bib-0030] Higginbottom, G. , Pillay, J. J. , & Boadu, N. Y. (2013). Guidance on performing focused ethnographies with an emphasis on healthcare research. Qualitative Report, 18(9), 1–16.

[jan16295-bib-0031] Jennings, N. , Lowe, G. , & Tori, K. (2021). Nurse practitioner locums: A plausible solution for augmenting health care access for rural communities. Australian Journal of Primary Health, 27(1), 1–5. 33508211 10.1071/PY20103

[jan16295-bib-0032] Jones, D. , George, C. , Hart, G. K. , Bellomo, R. , & Martin, J. (2008). Introduction of medical emergency teams in Australia and New Zealand: A multi‐centre study. Critical Care, 12(2), 1–8. 10.1186/cc6857 PMC2447594 18394192

[jan16295-bib-0033] Knott, E. , Rao, A. H. , Summers, K. , & Teeger, C. (2022). Interviews in the social sciences. Nature Reviews Methods Primers, 2(1), 73.

[jan16295-bib-0034] Koželj, A. , Šikić Pogačar, M. , Fijan, S. , Strauss, M. , Poštuvan, V. , & Strnad, M. (2021). Exploring the feelings of nurses during resuscitation—A cross‐sectional study. Healthcare (Basel, Switzerland), 10(1), 5. 35052169 10.3390/healthcare10010005 PMC8774964

[jan16295-bib-0035] Lincoln, Y. S. , & Guba, E. G. (1985). Naturalistic inquiry. SAGE.

[jan16295-bib-0036] Nilsen, P. , Neher, M. , Ellström, P. E. , & Gardner, B. (2017). Implementation of evidence‐based practice from a learning perspective. Worldviews on Evidence‐Based Nursing, 14(3), 192–199. 28281328 10.1111/wvn.12212

[jan16295-bib-0037] Noy, C. (2008). Sampling knowledge: The hermeneutics of snowball sampling in qualitative research. International Journal of Social Research Methodology, 11(4), 327–344.

[jan16295-bib-0038] NSW Ministry of Health . (2021). NSW health guide to the role delineation of clinical services. NSW Ministry of Clinical Innovation. https://www.health.nsw.gov.au/services/Publications/role‐

[jan16295-bib-0039] O'Reilly, K. (2012). Ethnographic methods (2nd ed.). Routledge.

[jan16295-bib-0040] Pahwa, M. , Cavanagh, A. , & Vanstone, M. (2023). Key informants in applied qualitative Health Research. Qualitative Health Research, 10497323231198796. 10.1177/10497323231198796 PMC10666509 37902082

[jan16295-bib-0041] Polit, D. F. , & Beck, C. T. (2017). Nursing research: Generating and assessing evidence for nursing practice (10th ed.). Wolters Klower.

[jan16295-bib-0042] Pulla, V. , & Carter, E. (2018). Employing interpretivism in social work research. International Journal of Social Work and Human Services Practice, 6(1), 9–14.

[jan16295-bib-0043] Reed, B. , & Bendall, J. (2015). Rural People's use of ambulances to reach emergency departments in potentially serious health emergencies: Identifying patterns of use and non‐use. Australasian Journal of Paramedicine, 12, 1–7.

[jan16295-bib-0044] Riley, K. , Middleton, R. , Wilson, V. , & Molloy, L. (2022). Voices from the ‘resus room’: An integrative review of the resuscitation experiences of nurses. Journal of Clinical Nursing, 31(9–10), 1164–1173. 34542206 10.1111/jocn.16048

[jan16295-bib-0045] Riley, K. , Wilson, V. , Middleton, R. , & Molloy, L. (2024). Examining the roles of rural nurses in resuscitation care: An ethnographic study. International Emergency Nursing, 73, 101404. 38325062 10.1016/j.ienj.2023.101404

[jan16295-bib-0046] Roper, J. M. , & Shapira, J. (2000). Ethnography in nursing research (Vol. 1). SAGE Publications.

[jan16295-bib-0047] Ryan, G. (2018). Introduction to positivism, interpretivism and critical theory. Nurse Researcher, 25(4), 41–49. 10.7748/nr.2018.e1466 29546962

[jan16295-bib-0048] Sariyer, G. , Ataman, M. G. , Sofuoğlu, T. , & Sofuoğlu, Z. (2017). Does ambulance utilization differ between urban and rural regions: A study of 112 services in a populated city, Izmir. Journal of Public Health, 25, 379–385.

[jan16295-bib-0049] Sedgwick, M. , & Pijl‐Zieber, E. M. (2015). New rural acute care nurses speak up: “We're it” but we're not ready. Journal for Nurses in Professional Development, 31(5), 278–283. 26381338 10.1097/NND.0000000000000188

[jan16295-bib-0050] Shenton, A. K. (2004). Strategies for ensuring trustworthiness in qualitative research projects. Education for Information, 22(2), 63–75.

[jan16295-bib-0051] Slattery, P. , Saeri, A. K. , & Bragge, P. (2020). Research co‐design in health: A rapid overview of reviews. Health Research Policy and Systems, 18(1), 1–13. 32046728 10.1186/s12961-020-0528-9 PMC7014755

[jan16295-bib-0052] Stahl, N. A. , & King, J. R. (2020). Expanding approaches for research: Understanding and using trustworthiness in qualitative research. Journal of Developmental Education, 44(1), 26–28.

[jan16295-bib-0053] Swain, J. , & King, B. (2022). Using informal conversations in qualitative research. International Journal of Qualitative Methods, 21, 16094069221085056.

[jan16295-bib-0054] Theobald, K. A. , Coyer, F. M. , Henderson, A. J. , Fox, R. , Thomson, B. F. , & McCarthy, A. L. (2020). Developing a postgraduate professional education framework for emergency nursing: A co‐design approach. BMC Nursing, 20(1), 43. 10.1186/s12912-021-00560-z PMC7953725 33712011

[jan16295-bib-0055] Welch, A. (2014). Sampling in qualitative research. In S. Jirojwong , M. Johnson , & A. Welch (Eds.), Research methods in nursing and midwifery: Pathways to evidence‐based practice (pp. 89–96). Oxford University Press.

[jan16295-bib-0056] Whiteing, N. , Barr, J. , & Rossi, D. M. (2022). The practice of rural and remote nurses in Australia: A case study. Journal of Clinical Nursing, 31(11–12), 1502–1518. 34396616 10.1111/jocn.16002

[jan16295-bib-0057] Wolf, L. , & Delao, A. M. (2013). Identifying the educational needs of emergency nurses in rural and critical access hospitals. The Journal of Continuing Education in Nursing, 44(9), 424–428. 23964675 10.3928/00220124-20130816-38

[jan16295-bib-0058] Won, Y. H. , & Kang, J. (2022). Development of a comprehensive model for the role of the rapid response team nurse. Intensive & Critical Care Nursing, 68, 103136. 34736834 10.1016/j.iccn.2021.103136

[jan16295-bib-0059] Yamada, N. K. , Kamlin, C. O. F. , & Halamek, L. P. (2018). Optimal human and system performance during neonatal resuscitation. Seminars in Fetal & Neonatal Medicine, 23, 306–311. 10.1016/j.siny.2018.03.006 29571705

[jan16295-bib-0060] Zanno, A. , Melendi, M. , Cutler, A. , Stone, B. , Chipman, M. , Holmes, J. , & Craig, A. (2022). Simulation‐based outreach program improves rural Hospitals' team confidence in neonatal resuscitation. Cureus, 14(9), e28670. 36196287 10.7759/cureus.28670 PMC9525099

